# Hearing loss in osteogenesis imperfecta patients

**DOI:** 10.1186/1687-9856-2015-S1-P68

**Published:** 2015-04-28

**Authors:** Lai Thu Ha, Vu Chi Dung, Can Thi Bich Ngoc, Bui Phuong Thao, Nguyen Ngoc Khanh, Luong Hong Chau

**Affiliations:** 1 Otolaryngology department, National Hospital of Pediatrics, Hanoi, Vietnam; 2 Department of Endocrinology, Metabolism and Genetics, National Hospital of Pediatrics, Hanoi, Vietnam; 3 Ear Department, Ear - Nose - Throat National Hospital, Hanoi, Vietnam

## 

Osteogenesis imperfecta (OI) is an inherited bone and connective tissue disorder associated with the lifelong occurrence of frequent fractures following even mild trauma. Hearing loss is frequently reported in patients with OI. Objective: to examine the ratio of hearing loss in children with OI, and the relationship between audiological findings and CT images of temporal bone in children with OI. Subject and methods: forty – two children aged 5 to 17 years with OI were included in the study. The patients have type A of tympano and were mesured thresold of hearing by play audiometry. CT imaging was performed in 8 cases as well. Imaging abnormalities were correlated with clinical phenotypes and severity of hearing loss deduced from audiograms. Results: Hearing loss of all etiologies was observed in 28.05 % of ears in studied OI patients. Sensorineural and mixed hearing loss was observed in 4.88% and conductive hearing loss was detected in 23.17% of ears. CT revealed bone – bridge image in the middle ear (10/16 ears), hypodense foci in the fissula ante fenestram (4/16 ears) and cochlear (2/16 ears), abnormal stape ( 5/16 ears). Conclusions: hearing loss in children with osteogenesis imperfecta is quite frequent. We have all type of hearing loss, but the conductive of hearing loss have highest ratio. The site of abnormal on temporal bone CT images in OI corresponds to presence and type of hearing loss determined by audiometry.

